# Heads or tails: investigating the effects of amphiphile features on the distortion of chiral nematic liquid crystal droplets†

**DOI:** 10.1039/d2tc05390j

**Published:** 2023-03-22

**Authors:** Lawrence W. Honaker, Jorik Schaap, Dennis Kenbeek, Ernst Miltenburg, Siddharth Deshpande

**Affiliations:** a Laboratory of Physical Chemistry and Soft Matter, Wageningen University & Research 6708 WE Wageningen The Netherlands siddharth.deshpande@wur.nl +31 (0)317 480 419

## Abstract

Liquid crystal-based sensing has fast become a growing field, harnessing the sensitivity of liquid crystals to their surroundings to provide information about the analytes present, including surface-active amphiphiles such as biological lipids. Amphiphiles can impart ordering to a liquid crystal and, in the case of chiral nematic liquid crystals (CLCs), distort the helical texture. The cause and degree to which this distortion occurs is not fully clear. In this work, the effects of different amphiphiles on the final colour textures as well as the pitch of chiral nematic liquid crystals are investigated. We find that the tails of amphiphiles and their orientation play a more important role in determining the final distortions of the liquid crystal by the direct interactions they have with the host, whereas the headgroups do not play a significant role in affecting these distortions. Our findings may find implications in designing CLC-based biosensors, where the tails will likely have more impact on the CLC response, while the headgroups will remain available for further functionalization without having significant effects on the signal readout.

## Abstract 2

The tails of amphiphiles play a dominant role than the heads when interacting with chiral nematic liquid crystal droplets and determining the final textures and distortions.

## Graphical abstract

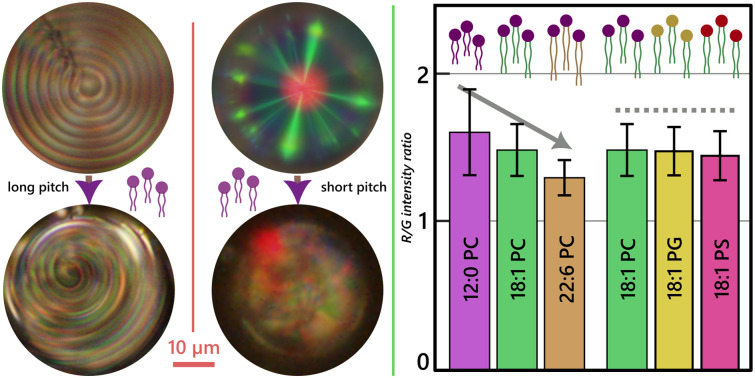

## 1 Introduction

Long known for their use in display applications,^1^ liquid crystals (LCs) have become increasingly popular for biological sensing applications. This is due to the high sensitivity of their alignment to the surroundings together with a clear optical feedback,^2,3^ indicating the presence of analytes that induce an alignment and ordering change.^4–14^ When chiral nematic (or cholesteric) liquid crystals (CLCs) – which have an additional helical modulation along with the long-range orientational order – are used, different textures can emerge.^15^ By using CLC materials with a pitch on the same order as the wavelength of visible light, this response can manifest itself in the form of strongly reflected colors that can be seen by the unaided eye.^16–19^ The mechanism of how the response is generated depends on what is being sensed: with gas sensing, often one sees a phase transition in the LC due to a depression of the clearing point temperature at which the liquid crystal phase becomes lost.^9,13,16,20^ On the other hand, amphiphile sensing typically looks at a change in the alignment and orientation of the LC induced by the adsorption of a surface-active molecule at the interface.^5,18,21–24^ Other variants of amphiphile adsorption can also use binding events between antibodies and antigens which disrupt the orientation of the LC to detect macromolecules and proteins,^6,12,25,26^ using a distortion or disruption in the texture to identify the presence or introduction of a target analyte.

**Fig. 1:**
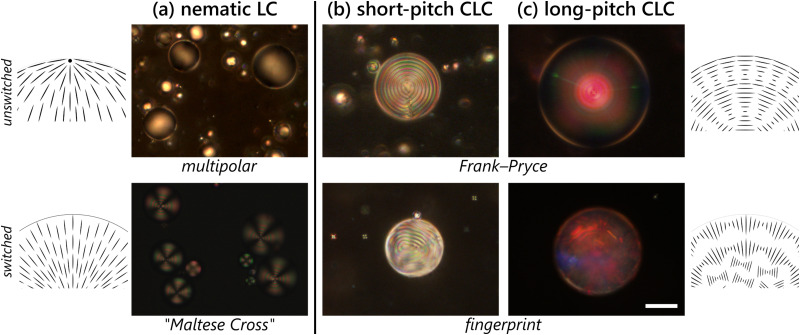
The amount of chiral dopant added to an LC sample affects the pitch of the material and thus the outcome of switching. Droplets of liquid crystals prepared from (a) pure 5CB (4-cyano-4′-pentylbiphenyl), a nematic phase; (b) 3% w/w chiral dopant CB15 ((*S*)-4-cyano-4′-(2-methylbutyl)biphenyl) mixed into 5CB; and (c) 35% w/w CB15 in E7. In all cases, droplets are stabilized by 0.2% w/w PVA (unswitched) and exposed to 1.0 mM sodium dodecyl sulfate (SDS) solution (switched). The textures in (a) are the characteristic polar planar (unswitched) and “Maltese Cross” homeotropic (switched) that we expect for a nematic liquid crystal. In (b), the characteristic Frank–Pryce texture consisting of rings with helical axis orthogonal to the surface of the droplet changes to a “fingerprint” in presence of surfactants, where the helical axis of the LC lies along the surface of the droplet instead with interference patterns from both hemispheres. In (c), the effects of the unswitched Frank–Pryce texture manifest in the form of central droplet reflections with blue-shifted inter-droplet reflections (red to green), while the switched textures provide a complex, scattering pattern with complex color signatures. The alignment induced by the homeotropic nematic^45^ and the Frank–Pryce texture in the chiral nematic^37^ have been shown to penetrate through the droplet. However, the degenerate anchoring associated with the planar nematic and fingerprint chiral nematic are not assumed to impart as strong ordering within the droplet bulk. Scale bar 25 μm.

A common question when using any LC-based sensors is how the output signal can be interpreted and translated into information about the system and what conditions can switch the LC alignment. Commonly, when using non-chiral nematic LCs, this is in the form of using the LC as a binary on–off switch, where a change in alignment^8,12,14,22,25–28^ or the loss of liquid crystalline ordering altogether^9,13,16,27^ will indicate the presence or absence of an event. Some chiral nematic LC materials will additionally function as an on–off switch, most commonly in quick-read temperature applications.^29–31^ It is, however, difficult to extract information about the analyte identity purely from the final texture of a nematic LC, save a few exceptions: for example, recent work has shown that specific information about the volatile compounds that are being sensed can be extracted based on the dynamics of the LC response^9,32^ or by attaching specific antibodies to the interface.^26^ CLCs and CLC-derived materials, however, can instead show gradients of response on their own^11,17,33–36^ owing to distortions induced in the helical structure that change the reflected color. These color changes are potentially more amenable to eventual sensing applications without targeting specific molecules, often able to be read out by the unaided eye^33,35^ and without the traditional crossed polarizer set-ups necessary with nematic LC materials. Based on our previous work on general biological sensing of amphiphilic molecules with CLC droplets,^24^ we wanted to further probe the effects of the structure of amphiphiles on the final orientation of the droplets and the resultant colors we see and what is ultimately responsible for creating the changes in texture. By using a CLC with a pitch on the order of the wavelength of light, the optics of the CLC orientation are manifested through visible colors^18,23,37^ owing to structural color that arises from Bragg reflection off the CLC helical structure.^38^ As shown in Fig. 1, by tuning the amount of chiral dopant present in the CLC (and, accordingly, the pitch), we can see diverse optical responses: (a) the non-chiral nematic LC exhibits a characteristic transition from a planar multipolar structure to a “Maltese Cross” upon switching in the presence of 1 mM sodium dodecyl sulfate (SDS). (b) A CLC with a visible long pitch *p*_0_ (order of μm, but significantly less than the droplet radius) initially shows a Frank–Pryce texture, with the helical axis oriented normal to the droplet surface, creating a characteristic“bull's eye” owing to the pseudolayering created by the helical modulation of the LC.^37,39,40^ In presence of 1 mM SDS, the droplet switches to a “fingerprint” texture where the helical axis instead sits tangentially along the droplet interface.^41,42^ (c) For CLCs with short pitches, comparable to the wavelength of visible light, the responses are manifested in colors in the form of a characteristic “starburst” associated with the Frank–Pryce texture and cross-droplet communication^18,37,43^ to form a multidomain, diffuse, “chicken-skin” pattern^18,23^ associated with the degeneracy of the CLC helix axis orientation at the interface.^44^ It has been previously hypothesized that a combination of surface effects, affecting the distribution of the amphiphile molecules at the interface, plus the distortion of the LC produced by the inclusion of the aliphatic tails into the LC (and the consequent changes to the helical twisting power of the chiral dopant used to generate the CLC phase^15,24,41^), is responsible for the evolution of the textures we see: in the case of pitches on the order of visible light, this is also reflected in changes of reflected colors. What is less clear is what aspects of the amphiphile structure are most responsible for the final textures generated. We wish to know which aspects of amphiphiles are particularly detectable using CLCs: can we, for example, determine if a headgroup is different, and/or can we distinguish between amphiphilic molecules with different tails?

In this work, we investigate how the properties of individual amphiphiles will distort a CLC and the effects on the output signal that result from this distortion by using a selection of homologous phospholipids and surfactants that differ in either the headgroups or the tails. We find that differences in adsorption caused both by head and tail effects at the interface affect the final textures, though in different manners: differences in the headgroup affect more the packing distribution of the lipids at the surface, while the tails themselves cause the distortion directly, with effects of the tails overall being much more prominent in governing the final configuration. We visualize this using both long-pitch CLC materials, where we can distinctly see the liquid crystalline helical ordering and how this changes upon the adsorption of amphiphiles, and with short-pitch CLC materials, where this distortion is instead manifested through changes in Bragg reflection and the colors we detect. With short-pitch CLC materials, we particularly look at the ratio of red-to-green (R/G) color channel intensities, finding that these do not significantly differ as a function of headgroup but that there are significant differences as a function of tails. These results can have implications in designing sensing technologies to directly use CLC materials to sense amphiphiles without the use of secondary detection methods.

## 2 Results and discussion

### 2.1 Amphiphile-induced distortions visualized by using long pitch chiral nematic materials

**Fig. 2:**
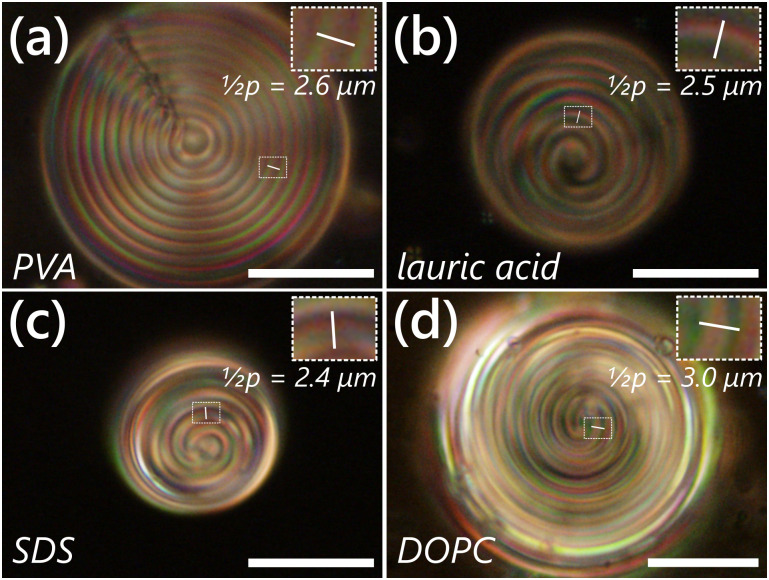
Using long pitch CLC materials can help us directly visualize how the pitch changes upon addition of amphiphiles. Droplets of a mixture of 3% w/w CB15 in 5CB were prepared in buffers containing (a) 0.02% w/w PVA, resulting in the characteristic Frank–Pryce texture associated with tangential LC/normal helical alignment visible at the droplet equator; and 1.0 mM each (b) lauric acid, (c) SDS, and (d) the phospholipid DOPC, where the LC is normally aligned and thus the helical modulation occurs tangentially along the LC surface, resulting in a characteristic “fingerprint” texture.^39^ Insets and white lines indicate a sample of how the pitch was determined, using a half pitch that was then doubled to obtain the final value. The final pitch values in Table 1 represent an average of at least 15 measurements. Images are reflection mode POM micrographs imaged between crossed linear polarizers. Scale bars 25 μm.

A natural question is whether or not we can actually visualize the distortion of the pitch due to the adsorption of amphiphiles and what effects the amphiphiles have on distorting the structure. To do so, we prepared mixtures of a longer pitch cholesteric and determined the pitch visually. We created a mixture of 3% w/w CB15 in the nematic LC host 5CB and exposed them to different solutions of amphiphiles, as shown in Fig. 2. The determination of the final pitch was achieved by measuring the spacing between the edges of the peaks, across one bright band, to get a half-pitch value which was then doubled to obtain the final equilibrium pitch. We determined the equilibrium pitch (in the Frank–Pryce texture) of this mixture (Fig. 2(a)) to be *p*_0_ = 5.2 ± 0.2 μm. Upon exposure to various surfactants, we saw a clear change in the pitch values in most of the samples, although to different extents, as shown in Table 1. The pitch of the samples exposed to lauric acid and SDS (sodium dodecyl sulfate) decreased from the equilibrium value, but they were found to be quite similar to each other (4.9 ± 0.1 μm and 4.8 ± 0.1 μm, respectively). The presence of CTAB affected the pitch value the least, with the pitch we determined being quite similar to the equilibrium pitch *p*_0_. We do note, however, that CTAB has a Krafft temperature of 24 °C and can have solubility issues at room temperature.^46^ This means it may not be able to adsorb to the interface as strongly, though its precise effect is not clear.

**Table 1:** Measured pitches of a 3% w/w CLC mixture with different adsorbed amphiphiles. Pitch values represent mean ± standard deviation of measurements obtained from at least 15 droplets in each case

Amphiphile	State	Pitch (μm)
PVA	Frank–Pryce	5.2 ± 0.2
lauric acid	Fingerprint	4.9 ± 0.1
SDS	Fingerprint	4.8 ± 0.1
CTAB	Fingerprint	5.1 ± 0.1
l-DLPC	Fingerprint	5.7 ± 0.1
l-DOPC	Fingerprint	6.1 ± 0.2

We observed a significant increase in the pitch value (5.7 ± 0.1 μm) in presence of the phospholipid DLPC (1,2-dilauroyl-*sn*-glycero-3-phosphocholine, l-DLPC, with two 12-carbon tails, analogous to SDS and lauric acid). We notice that there are two factors playing a role here: first, there are two tails, rather than one, which impart a distortion in the LC. Second, DLPC itself has an inherent permanent chirality. Since the CLC is generated by the inclusion of a chiral dopant into a non-chiral liquid crystal material, it is conceivable that the addition of other chiral materials can cause much stronger distortions in the LC, either by increasing or decreasing the pitch dependent on how much the amphiphile “cooperates” with the dopant.^41^ Thus, despite the differences in certain pitch values being close to the optical resolution (approx. 400 nm), we could see significant pitch differences in certain cases and at least semi-quantitative shifts for others. In the case of using DOPC (1,2-dioleoyl-*sn*-glycero-3-phosphocholine, l-DOPC, with two 18-carbon tails), the calculated pitch value (6.1 ± 0.2 μm) was found to be quite different from DLPC, despite the similar heads, hinting to a stronger role of amphiphile tails in distorting the LC compared to the headgroups (much like how both SDS and lauric acid provided quite similar pitch values), which we will explore shortly with short-pitch CLCs.

While beyond the scope of this work, it is conceivable that the use of lipids with different chirality (for example, d-DLPC rather than the l-DLPC we use here) will have a different effect, perhaps contracting the pitch rather than expanding it in combination with the chiral dopant. This chirality can be considered an aspect of the tail rather than the headgroup, though, owing to the location of the chiral center. Due to a lack of ready availability of oppositely handed lipids, such a study is not within the scope of this paper, but would prove interesting for further investigation.

### 2.2 Effects of headgroups on short-pitch chiral nematics

For this stage of the work, we used as the CLC material a red-reflecting mixture of 35% w/w CB15 chiral dopant (*S*)-4-cyano-4′-(2-methylbutyl)biphenyl, Fig. S1(b), ESI†) in the eutectic liquid crystal blend RO-TN 407. This produces a CLC material with broad temperature stability of the chiral nematic phase and a central reflection of *λ* ∼ 650 nm. This CLC mixture exhibits a stable liquid crystal phase over long periods without visible micro- or macroscopic phase separation. To investigate the effects of headgroups on the distortion of a CLC, we selected three phospholipids with different headgroups but with identical aliphatic tails: 1,2-dioleyoyl-*sn*-glycero-3-phosphocholine (DOPC), a zwitterionic lipid with a neutral terminal group at pH 7.4; 1,2-dioleyoyl-*sn*-glycero-3-phospho-(1′-*rac*-glycerol) (DOPG), which has a small headgroup that is negatively charged at pH 7.4; and 1,2-dioleyoyl-*sn*-glycero-3-phospho-l-serine (DOPS), which has a negatively charged l-serine terminal headgroup. Because the tails here are identical, in principle, any differences in the textures we see are likely a result of the headgroups. Additionally, two of the headgroups (PG and PS) are similar to each other compared to the PC head group, allowing further comparison within the optical responses. We prepared the lipid samples at 1.0 mM concentrations in Tris pH 7.4 buffer to maintain a constant, physiologically relevant pH. These three lipids are used at above the manufacturer-reported transition temperatures, and they all are easily prepared in aqueous dispersions at the relevant concentrations.

**Fig. 3:**
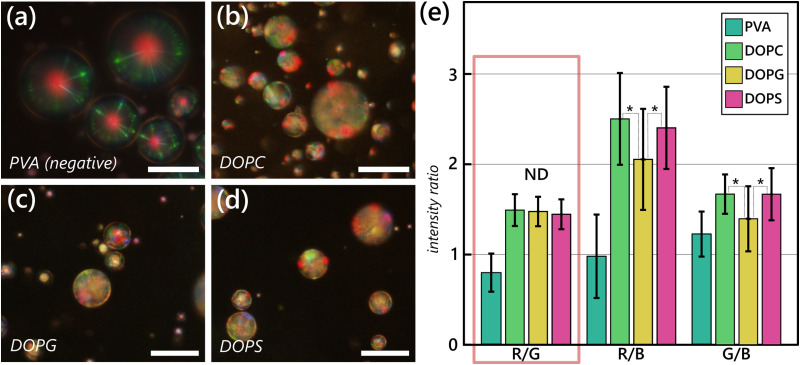
Statistically significant differences are not particularly apparent in all samples of liquid crystal droplets exposed to lipids with different headgroups and identical tail lengths. Reflection mode micrographs taken of samples of droplets of a red-reflecting CLC mixture (35% w/w CB15 in RO-TN 407) prepared in (a) 0.02% w/w PVA solution, producing a Frank–Pryce unswitched texture; and 1.0 mM each of (b) DOPC; (c) DOPG; and (d) DOPS solutions. (e) An analysis of the color ratios observed over a population size *N* ≥ 180 for each lipid sample and *N* ≥ 90 for PVA. No statistically significant differences (ND, *p* ≥ 0.001) are observed to be present between DOPC, DOPG, and DOPS on the R/G ratio. PVA results are shown as a comparison, showing that the samples appear qualitatively and quantitatively different from the samples exposed to lipids. Some statistically significant differences (indicated with asterisks, *p* ≤ 0.001)) are observable on the R/B and G/B channels. Scale bars in (a)–(d) 25 μm. Error bars in (e) indicate the standard deviation of the mean. Data for PVA are reproduced from Honaker *et al*^24^ under a CC-BY 4.0 License.

Sample micrographs of the textures we see along with a statistical analysis are presented in Fig. 3. We used a simple but effective color analysis that we recently reported^24^ to distinguish between the obtained optical responses. The analysis is based on the ratios of the three primary color channels (R/G, R/B, and G/B) to distinguish between population-level response to different amphiphiles. In this work, to establish if differences are present in populations, we paid particular attention to the R/G ratios, as red and green are the most prominent colors in the Frank–Pryce textures of the unswitched droplets, as shown in Fig. 2(d). Since red and green are the main colors we expect to see from Bragg reflection, these should be dominant in the spectrum. Analyzing the color ratios, we notably find that there are no statistically significant differences (*p* ≤ 0.001) between most of the color ratios, and particularly between the R/G ratios (see Table S1, ESI† for more details). We also analyzed the dependence on the droplet size (Fig. S2, ESI†), with all three ratios being largely size-invariant. The surface coverages of these three lipids are comparable to each other. All headgroups are overall quite similar in size, and this could account for the strong similarity of their color signals, but particularly the similarity in comparing the R/G ratios (with very few statistically significant differences found). This data is congruent with what we observed in long-pitch CLC materials, where two surfactants with different headgroups but identical tails (SDS and lauric acid) produced the same final pitch. This overall suggests to us that the headgroups may have some impact, but not a significant one, on the final textures we see, as we hypothesize graphically in Fig. 5. We do note that, in samples prepared from DOPG and DOPS, not all the droplets were fully switched from the Frank–Pryce texture characteristic of tangential/planar LC anchoring^18,47^ (as shown in Fig. 1(d)) to the multidomain fingerprint texture characteristic of surfactant adsorption.^24,48^ There appeared to be some effects of size, where extremely small (radius less than 6 μm and excluded from our analysis) droplets were not effectively switched. In the case of DOPC, however, all the observed droplets were consistently fully switched. This may be a consequence of either an inability of DOPS and DOPG to pack well at the LC–water interface, especially in comparison to DOPC, or that the micelles we form of DOPC more readily “break” and adsorb at the interface, while DOPG and DOPS could form more stable micelles that do not preferentially adsorb to the interface and instead stabilize the droplets much in the same way that proteins or polymer stabilizers alone can.

### 2.3 Effects of different tails on short-pitch chiral nematics

We also sought to investigate the effects of lipid tails on the final textures on CLC droplets, using lipids with identical headgroups but tails of different length and rigidity and the same liquid crystal mixture we used for the headgroup study (35% CB15 in RO-TN 407). For this, we chose to use series of phosphocholine headgroup lipids with differing tails: DLPC, which has two fully saturated 12-carbon tails; 1,2-dipalmitoyl-*sn*-glycero-3-phosphocholine (DPPC), with saturated 16-carbon tails; DOPC, which has 18-carbon tails, each with a *cis* bond at the ninth carbons; and 1,2-didocosahexaenoyl-*sn*-glycero-3-phosphocholine (22:6 PC), which has two 22-carbon tails that each have six unsaturated *cis* bonds at the fourth, seventh, tenth, thirteenth, sixteenth, and nineteenth carbons. Because the headgroups here are identical, we expect the packing of the amphiphiles at the interface of the LC to be similar; thus, any differences in distortions should be directly an effect of the tails.

**Fig. 4:**
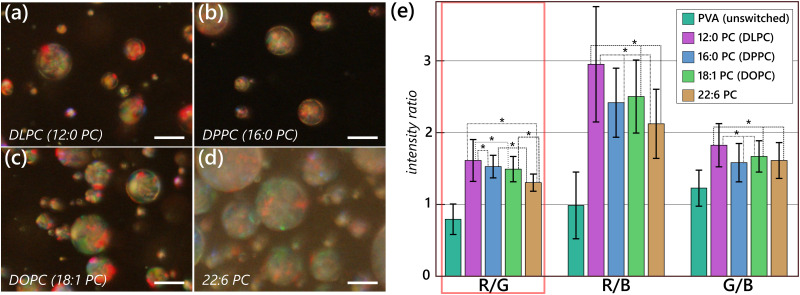
Statistically significant differences can become apparent between liquid crystal samples exposed to lipids with identical headgroups and differing tail lengths. (a)–(d) Representative reflection mode micrographs taken of droplets of a red-reflecting CLC mixture (35% w/w CB15 in RO-TN 407) prepared in solutions containing 1.0 mM (a) DLPC (12:0 PC); (b) DPPC (16:0 PC); (c) DOPC (18:1 PC); and (d) 22:6 PC. (e) A statistical analysis of the color ratios observed over a population size *N* ≥ 180 for each lipid sample and *N* ≥ 90 for PVA. Data for DOPC are replicated from Fig. 3(a) for ease of comparison, and data for PVA are replicated from Honaker *et al*^24^ under a CC-BY 4.0 License. Statistically significant differences (*p* ≤ 0.001) are indicated in the graph by connected bars with an asterisk. In case of the R/G ratio, save for the combination between DPPC and DOPC, all four lipids are statistically significantly different from each other and show a clear trend. Several differences are observable on the other channels, but without any trend. Scale bars in (a)–(d) 25 μm. Error bars in (e) indicate the standard deviation of the mean.

Fig. 4 presents sample micrographs of the four homologous lipids, along with a color ratio analysis. Unlike with the cases of using DOPS and DOPG, all the droplets visible within the field of view were fully switched. We observed more appreciable differences than those between headgroups and the differences were statistically significant between all of the samples, and especially on the R/G ratio, where statistically significant differences can be found between all four samples. We also observe a trend with respect to the R/G ratio, with a decrease in its value (meaning more green overall) with increasing tail length. A direct correlation of length to ratio cannot be drawn from this data, as there also may be effects due to saturation of the lipid tails (and thus the tail rigidity). For the other color ratios, there is no clear trend in signals as the tail length increases, though there are at least some statistically significant differences between the other ratios.

**Fig. 5:**
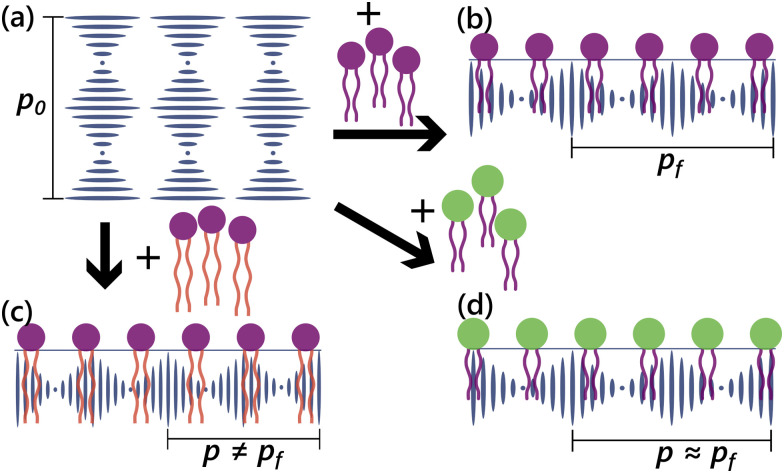
Changes to the different parameters of the amphiphile structure will have different consequent effects on the pitch of the CLC material.(a) The base CLC texture with equilibrium pitch *p*_0_ is planarly aligned at the interface, creating a helix oriented normally to the surface. (b) The addition of a surfactant or amphiphile will distort the pitch (*p*_f_), which may not be the same as *p*_0_. (c) Addition of a surfactant with different tails from (b) will more strongly distort the LC, producing a pitch change that strongly differs from the equilibrium switched pitch *p*_f_ (*p* ≠ *p*_f_) (d) A difference in the headgroup, however, does not strongly change the resultant switched pitch (*p*–*p*_f_), though we can still expect slight differences as we demonstrate with long-pitch CLCs. When this propagates into short-pitch cholesterics, changes in fingerprint pitch can additionally propagate into a change in the reflected colors. Schematic not to scale.

Particularly when compared to the minimal differences caused by headgroups (Fig. 3), the tails look to generally have stronger, significantly different effects on the distortion of the LC. This is likely because the headgroups will simply affect the distribution of lipids on the interface: if similarly sized headgroups were present, then differentiation between the different cases can become more difficult, as seen in the similarities in signal between DOPC, DOPG, and DOPS. In contrast, the tails directly induce distortions in the LC structure through their insertion into the CLC at the LC–water interface, interfering with the twist distortion created by the chiral dopant and the magnitude of which is dependent on the properties of the tails (rigidity and length). This is also in agreement with our results for the long-pitch CLC materials, as presented in Table 1, where identical tails (SDS and lauric acid) produced similar final pitch values, but identical headgroups (DLPC and DOPC) gave quite different distortions. These data suggest to us that the tail is the more determining factor in distorting the LC, particularly at the surface where the Bragg reflection is strongest and colors most visible. We expect that the main part of the optical reflection from the CLC will come from the surface.^36^ This is schematically represented in Fig. 5. With the cholesteric pitches at which we are working, the change in pitch will directly translate into a shift in reflected colors. We do note that, between different samples of 22:6PC, we did see differences in the responses induced by a freshly prepared sample compared to the same sample used the following day. This is likely due to 22:6PC being highly unsaturated (six double bonds on each of the tails) compared to other lipids. These double bonds could undergo hydrolysis reactions while in solution and thus give different responses from one day to the next. These effects, however, were not observed with the other lipids, likely due to either an absence or paucity of double bonds.

We also compared the head and tail groups not directly compared in Fig. 3 and 4 to each other (that is, comparing each of DLPC, DPPC, and 22:6 PC to DOPG and DOPS), the results of which can be seen in Table S1 (ESI†). In general, we do see statistically significant (*p* ≤ 0.001) differences between all groups, though not between DPPC and DOPG, much like the case of DPPC and DOPC. Because both headgroups and tail groups are varied in each of these cases, directly comparing these groups to each other is difficult; however, we do see that these groups are generally distinguishable from each other at the statistical significance level we use. These comparisons almost invariably involve a different tail, which further strengthens our hypothesis of the importance of the differences in tail being a primary determining factor in being to distinguish between amphiphiles with bare, unfunctionalized CLCs alone.

## 3 Conclusion

In this article, we have looked at how the structural differences in amphiphiles play a role in determining the final fingerprint textures that develop in a cholesteric liquid crystal material. We used both long-pitch and short-pitch micron-sized droplets and a wide variety of amphiphiles, in particular lipids, followed by a population-level analysis of the optical response. We conclude that the most prominent differences in the final textures (and optical signatures for short-pitch CLC materials) arise due to the properties of the tails rather than the headgroups. As indicated in Fig. 5, we expect that the amphiphile tails embed themselves into the CLC interior, contributing on their own to the distortions of the final CLC texture, while the main differences imparted by different headgroups arise primarily from how well they are packed at the surface and how well the amphiphiles are thus aligned at the LC–water interface. When working with chiral pitches closer to the order of the wavelengths of visible light, these subtle shifts in pitch can result in considerable changes in reflected colors and the optical responses of the CLC material.

Apart from the bettered understanding of how amphiphiles interact with CLC materials, this work does have implications in designing sensors based on CLCs. Since headgroups have much less of a significant effect compared to tails, this means the amphiphiles which adsorb at the interface can be readily functionalized, such as with biotinylation^26^ or fluorophore tags^24^ without having significant effects on the readouts we can expect to see. One of the directions of our future work will look to investigate effects of lipids and other amphiphiles, such as fatty acids, working together, seeing what signals can develop in the presence of multiple amphiphiles simultaneously. We will also look to investigate effects of chirality of amphiphiles, such as by using lipids or chiral dopants of opposite handedness, to see if chirality of the lipids play a role in the distortion of the texture. Other directions we could look to explore in the future might involve pattern detection and recognition, determining whether or not specific defects and patterns arise in the CLC material that can be used.

## 4 Materials and methods

### 4.1 Materials

The phospholipids 1,2-dilauroyl-*sn*-glycero-3-phosphocholine (DLPC); 1,2-dipalmitoyl-*sn*-glycero-3-phosphocholine (DPPC); 1,2-dioleyoyl-*sn*-glycero-3-phosphocholine (DOPC); 1,2-didocosahexaenoyl-*sn*-glycero-3-phosphocholine (22:6 PC); 1,2-dioleyoyl-*sn*-glycero-3-phospho-(1′-*rac*-glycerol) (DOPG); and 1,2-dioleyoyl-*sn*-glycero-3-phospho-l-serine (DOPS) in chloroform were sourced from Avanti Polar Lipids. Trizma base (tris(hydroxymethyl)aminomethane), and Trizma HCl (tris(hydroxymethyl)aminomethane hydrochloride) were sourced from Sigma Aldrich and combined in ultrapure water to obtain a buffer with pH 7.4 (Tris 7.4). Poly(vinyl alcohol) (*M*_w_ = 13–23 kDa, 87–89% hydrolyzed), sodium dodecyl sulfate (SDS, 99%), and hexadecyltrimethylammonium bromide (CTAB) were obtained from Sigma Aldrich. Lauric acid (99%) was sourced from Acros Organics. A chiral nematic liquid crystal mixture consisting of 35% w/w CB15 ((*S*)-4-cyano-4′ -(2-methylbutyl)biphenyl, Synthon GmbH) mixed into the eutectic liquid crystal blend RO-TN 407 (F. Hoffman–La Roche) was graciously provided to us by the University of Luxembourg. We additionally used the nematic liquid crystal 5CB (4-cyano-4′-pentylbiphenyl, 95%+, Tokyo Chemical Industry) and the eutectic nematic liquid crystal blend E7 (51% 5CB, 25% 4-cyano-4′-heptylbiphenyl, 16% 4-cyano-4′-octyloxybiphenyl, and 8% 4-cyano-4′-pentyl-*p*-terphenyl^49^; Synthon GmbH) to create different CLC mixtures with variable pitch. All chemicals were used without further purification. The structures of the chemicals used (where known) are presented in Fig. S1 (ESI†).

### 4.2 Experimental protocols

#### 4.2.1 Droplet generation and image acquisition

For lipids, sufficient quantities of phospholipids in chloroform were pipetted into clean glass vials to generate a concentration of 1.0 mM lipids in buffer solution. The chloroform was dried from the sample under vacuum, yielding a dried lipid film. The films were then hydrated with Tris 7.4 buffer solution at room temperature with occasional vortexing overnight. The lipid buffer solutions were then twice extruded through a 0.2 μm PVDF or PTFE filter to obtain a clear, optically transparent solution. Lipid buffer solutions were used within a week of preparation.

To produce droplets, in a clean Eppendorf vial, 3 μl of the CLC mixture was pipetted into 500 μl amphiphile buffer. The vial was then alternately shaken vigorously manually and mixed with a vortex mixture to obtain a cloudy dispersion of droplets. Immediately after producing the dispersion, ∼2.5–10 μl dispersion was pipetted onto a clean, untreated borosilicate glass microscopy slide and imaged using an Olympus BX60 polarizing optical microscope equipped with a DT70 color camera in reflection mode. Prepared slides then were left to stand on the microscope briefly before imaging to allow the droplets time to settle.

#### 4.2.2 Pitch measurements with long-pitch cholesterics

After being prepared in solution, a droplet of the dispersion of the long-pitch CLC material in buffer was pipetted on a clean microscopy slide and micrographs in reflection mode polarizing optical microscopy were acquired. These micrographs were then imported into FIJI (ImageJ) and the pitch determined manually by measuring the distance between the stripes indicating the helical modulation of the director, either located at the droplet equatorial plane (for the tangentially aligned/Frank–Pryce texture CLC materials) or on the droplet surface (for the switched droplets). The pitch was determined with manual measurements in FIJI and calculated as the average from at least 15 droplets.

#### 4.2.3 Color channel analysis

The acquired images were loaded into FIJI (ImageJ) and converted into 8-bit grayscale images. To smooth the data, a Gaussian blur with filter size 4 was applied. Images were thresholded to obtain binary images showing the liquid crystal droplets. To identify individual droplets, we ran a watershed transform on each binary image, obtaining a labeled image. To obtain the particles from this image, we took all regions with a minimum size and a circularity constraint. All obtained ROIs from the watershed transform were checked manually and removed in case of incorrect detection. LC droplets that were not recognized by the automatic analysis had their ROIs manually drawn.

To obtain the color ratios, the sum of intensities of the respective color channels was taken for each identified LC droplet using MATLAB (version 2022a). Droplets with radius smaller than 6.3 μm (too small for a robust analysis and also many times out-of-focus) and larger than 47.5 μm (no clear alignment change) were excluded from the analysis. Droplets where the color signals were not easily distinguishable (primarily due to not being in focus), and droplets where the alignment was not well-defined (primarily the case for larger droplets) were also discarded. The sum of intensity was then divided for each color channel (*e.g.*
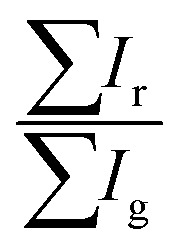
) and the color ratios were obtained. The histograms are averages of multiple sets of data collected over different days. We determined whether the data sets were statistically significantly different from each other by using a Tukey Honestly Significant Difference (HSD) test, the results of which are reported in Table S1 (ESI†), using a *p*-value of 0.001 as the cut-off for statistical significance.

## Author contributions

Conceptualization – L. W. H. & S. D.; data curation – L. W. H. & J. S.; formal analysis – L. W. H., J. S., & E. M.; funding acquisition – S. D.; investigation – L. W. H., J. S., E. M., & D. K.; methodology – L. W. H., J. S., D. K., & E. M.; project administration – S. D.; supervision – L. W. H. & S. D.; writing – original draft – L. W. H., J. S., & S. D.; writing – review & editing – L. W. H., J. S., & S. D. All authors have read and agreed to the published version of the manuscript.

## Conflicts of interest

There are no conflicts to declare.

## Supplementary Material

TC-011-D2TC05390J-s001
