# Correction: Heads or tails: investigating the effects of amphiphile features on the distortion of chiral nematic liquid crystal droplets

**DOI:** 10.1039/d3tc90080k

**Published:** 2023-04-18

**Authors:** Lawrence W. Honaker, Jorik Schaap, Dennis Kenbeek, Ernst Miltenburg, Siddharth Deshpande

**Affiliations:** a Laboratory of Physical Chemistry and Soft Matter, Wageningen University & Research 6708 WE Wageningen The Netherlands siddharth.deshpande@wur.nl +31 (0)317 480 419

## Abstract

Correction for ‘Heads or tails: investigating the effects of amphiphile features on the distortion of chiral nematic liquid crystal droplets’ by Lawrence W. Honaker *et al.*, *J. Mater. Chem. C*, 2023, **11**, 4867–4875, https://doi.org/10.1039/d2tc05390j.

The authors regret an error in Fig. 1 of the published article, in which the labels for panels (b) and (c) were inadvertently exchanged. The corrected Fig. 1 is shown below (the figure caption is unchanged).

**Fig. 1:**
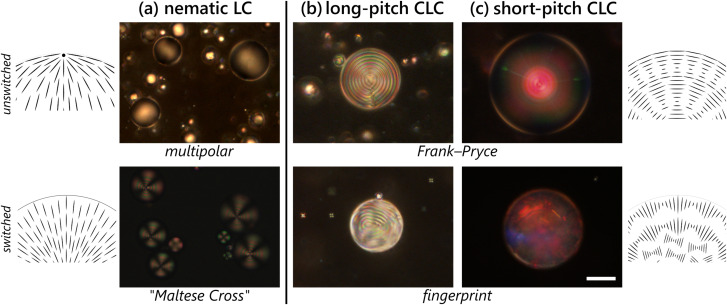
The amount of chiral dopant added to an LC sample affects the pitch of the material and thus the outcome of switching. Droplets of liquid crystals prepared from (a) pure 5CB (4-cyano-4′-pentylbiphenyl), a nematic phase; (b) 3% w/w chiral dopant CB15 ((*S*)-4-cyano-4′-(2-methylbutyl)biphenyl) mixed into 5CB; and (c) 35% w/w CB15 in E7. In all cases, droplets are stabilized by 0.2% w/w PVA (unswitched) and exposed to 1.0 mM sodium dodecyl sulfate (SDS) solution (switched). The textures in (a) are the characteristic polar planar (unswitched) and “Maltese Cross” homeotropic (switched) that we expect for a nematic liquid crystal. In (b), the characteristic Frank–Pryce texture consisting of rings with helical axis orthogonal to the surface of the droplet changes to a “fingerprint” in presence of surfactants, where the helical axis of the LC lies along the surface of the droplet instead with interference patterns from both hemispheres. In (c), the effects of the unswitched Frank–Pryce texture manifest in the form of central droplet reflections with blue-shifted inter-droplet reflections (red to green), while the switched textures provide a complex, scattering pattern with complex color signatures. The alignment induced by the homeotropic nematic^45^ and the Frank–Pryce texture in the chiral nematic^37^ have been shown to penetrate through the droplet. However, the degenerate anchoring associated with the planar nematic and fingerprint chiral nematic are not assumed to impart as strong ordering within the droplet bulk. Scale bar 25 μm.

The Royal Society of Chemistry apologises for these errors and any consequent inconvenience to authors and readers.

## Supplementary Material

