# Supplementary material for: Heads or tails: investigating the effects of amphiphile features on the distortion of chiral nematic liquid crystal droplets
Source: J Mater Chem C Mater. 2023 Mar 22;11(14):4867–75. doi: 10.1039/d2tc05390j (PMC10077502; doi:10.1039/d2tc05390j)
Supplement: TC-011-D2TC05390J-s001 [file TC-011-D2TC05390J-s001.pdf]

Electronic Supplementary Material (ESI) for Journal of Materials Chemistry C.
This journal is © The Royal Society of Chemistry 2023

Supporting Information for "Heads or Tails: Investigating the Effects of Amphiphile Features on the Distortion of Chiral Nematic Liquid Crystal Droplets"

Lawrence W. Honaker, Jorik Schaap, Dennis Kenbeek, Ernst Miltenburg, and Siddharth Deshpande
Laboratory of Physical Chemistry and Soft Matter, Wageningen University & Research, 6708 WE Wageningen, The Netherlands

A Chemical Structures

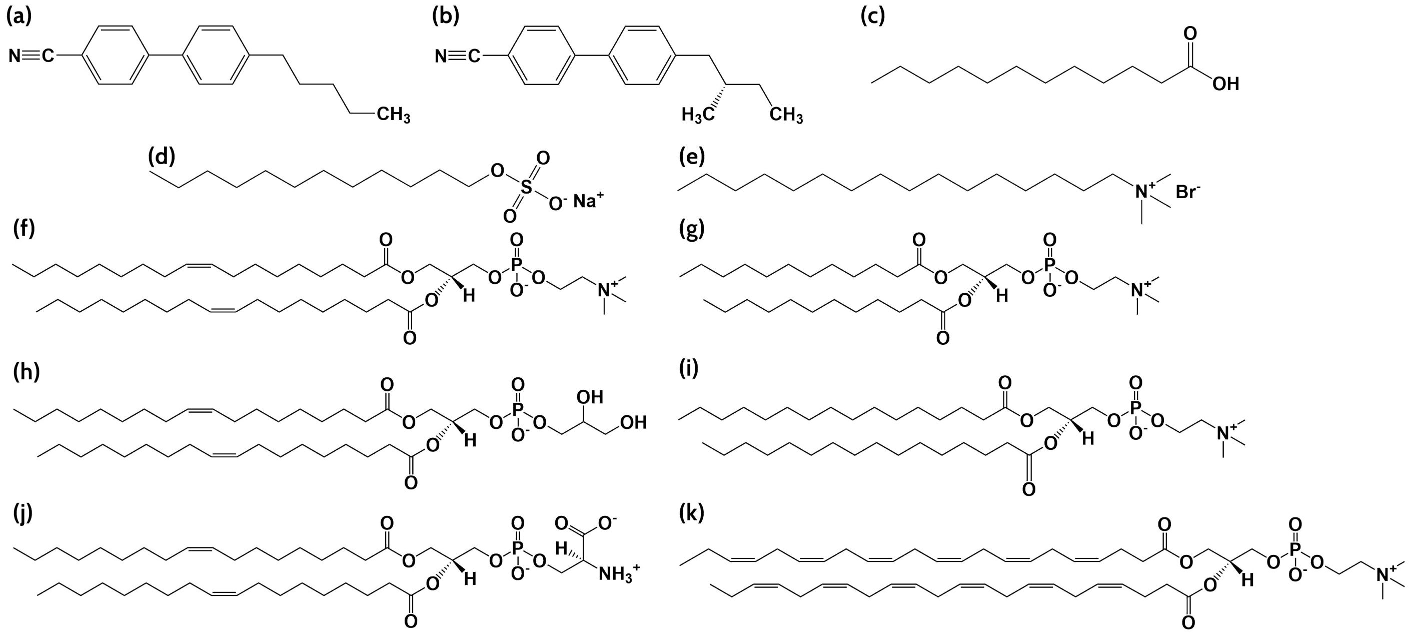

Fig. S1 Structures of the main compounds used in this study. (a) 4-cyano-4′-pentylbiphenyl (5CB), a room-temperature pure nematic liquid crystal; (b) (*S*)-4-cyano-4′-(2-methylbutyl)biphenyl (CB15), a chiral dopant used to produce the chiral nematic LC phase; (c) lauric acid (dodecanoic acid); (d) sodium dodecyl sulfate (SDS); (e) hexadecyltrimethylammonium bromide (CTAB); (f) 1,2-dioleyoyl-*sn*-glycero-3-phosphocholine (DOPC); (g) 1,2-dilauroyl-*sn*-glycero-3-phosphocholine (DLPC); (h) 1,2-dioleyoyl-*sn*-glycero-3-phospho-(1′-*rac*-glycerol) (DOPG); (i) 1,2-dipalmitoyl-*sn*-glycero-3-phospho-choline (DPPC); (j) 1,2-dioleyoyl-*sn*-glycero-3-phospho-L-serine (DOPS); (k) 1,2-didocosahexaenoyl-*sn*-glycero-3-phosphocholine (22:6 PC).

B Data Analysis

B.1 Effects of Droplet Size on Color Ratios

In Figure S2, we present an analysis of the effects of size on the color ratios of the droplets. Much like reported in our previous work[24], we find no effects on size for all the three color ratios.

B.2 Statistics

To determine whether the differences were statistically significant, we performed Tukey Honestly Significant Difference (HSD) tests using Matlab (version 2022a). The results of these are presented in Table S1. For completeness, we additionally report the data for the secondary comparisons that are not represented in Figures 3 and 4, finding that there are visible statistically significant differences between most of the other groups we do not compare, though there are differences in both heads and tails in each of these cases. All the lipid groups (both heads and tails) are simultaneously compared.

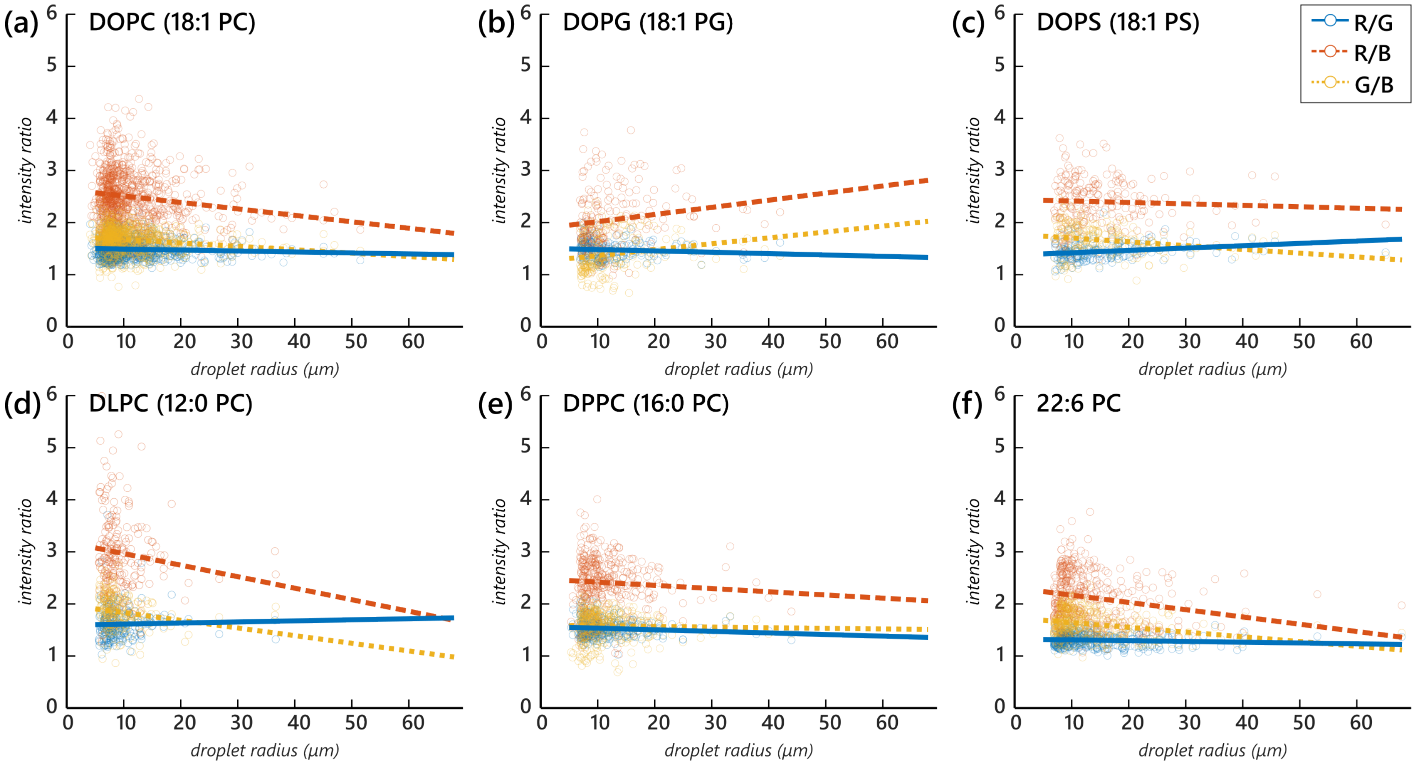

Fig. S2 The color ratios are strongly size invariant. A scatter plot of the effects of droplet size on color ratios (R/G ratio in blue, R/B ratio in orange, and G/B ratio in yellow)) for (a) DOPC, (b) DOPG, (c) DOPS, (d) DLPC, (e) DPPC, and (f) 22:6 PC on the size range from 6.3 μm) to 47.5 μm. We find no appreciable trend as a function of droplet size in any of the intensity ratios for all the six samples.Trendlines represent a linear regression across the interval with $R^2 < 0.05$ in all cases, emphasizing a very weak correlation.

Table S1 Statistical comparisons of the lipids, underlying the data presented in Figures 3 and 4. Highly statistically significant differences ($p \leq 0.001$) are indicated with *. Higher values ($p > 0.001$) were marked as no significant difference (ND). Values $p < 10^{-20}$ are used to indicate a p-value of 0 obtained in the analysis, *i.e.*, an extremely low *p* value.

Amphiphile A	Amphiphile B	*p* value	Verdict	Dataset
DOPC	DOPG	0.876	ND	Figure 3
DOPC	DOPS	0.0133	ND	
DOPG	DOPS	0.503	ND	
DLPC	DPPC	1.22×10^{-6}	*	Figure 4
DLPC	DOPC	8.27×10^{-17}	*	
DLPC	22:6 PC	$< 10^{-20}$	*	
DPPC	DOPC	0.0243	ND	
DPPC	22:6 PC	$< 10^{-20}$	*	
DOPC	22:6 PC	$< 10^{-20}$	*	
DLPC	DOPG	1.58×10^{-13}	*	Additional comparison
DLPC	DOPS	1.64×10^{-20}	*	
DPPC	DOPG	0.0145	ND	
DPPC	DOPS	3.68×10^{-6}	*	
DPPC	DOPG	$< 10^{-20}$	*	
DPPC	DOPS	1.06×10^{-19}	*	
